# Demographic differences in use of household tap water in a representative sample of US adults, FallStyles 2019

**DOI:** 10.2166/wh.2021.118

**Published:** 2021-12

**Authors:** Kayla Vanden Esschert, Catherine E. Barrett, Sarah A. Collier, Amanda G. Garcia-Williams, Elizabeth Hannapel, Jonathan S. Yoder, Katharine M. Benedict

**Affiliations:** a Waterborne Disease Prevention Branch, Division of Foodborne, Waterborne, and Environmental Diseases, National Center for Emerging and Zoonotic Infectious Diseases, Centers for Disease Control and Prevention, Atlanta, GA 30329, USA; b Epidemiology and Statistics Branch, Division of Diabetes Translation, National Center for Chronic Disease Prevention and Health Promotion, Centers for Disease Control and Prevention, Atlanta, GA 30329, USA; c Respiratory Diseases Branch, Division of Bacterial Diseases, National Center for Immunization and Respiratory Diseases, Centers for Disease Control and Prevention, Atlanta, GA 30329, USA

**Keywords:** aerosolized water, drinking water, environmental health, household water exposures, tap water, waterborne disease

## Abstract

Tap water that is safe to consume may cause respiratory illness (e.g., Legionnaires’ disease) when water conditions allow for proliferation and aerosolization of biofilm-associated pathogens. This study assessed household tap water consumption, exposure to aerosolized tap water, and associated demographics. A nationally representative FallStyles survey administered by Porter Novelli Public Services was sent to 4,677 US adult panelists in October 2019. There were 3,624 adults who completed the survey (77.5% response rate). Respondents were asked about self-reported use of household tap water for consumption (i.e., drinking, rinsing produce, or making ice) and use through water-aerosolizing devices (e.g., showerheads, humidifiers). Demographics included gender, age, race/ethnicity, education, income, region, and health status. Weighted analyses using complex sample survey procedures were used to assess tap water exposure by route and demographics. Most US adults are exposed to aerosolized tap water through showering (80.6%), and one in five are exposed through other water-aerosolizing devices (20.3%). Consumption and showering were greatest among older, White, higher educated, and higher-income adults. Aerosolized tap water can transmit waterborne pathogens and cause respiratory illness, especially among older age groups and people with weakened immune systems. These results will help target health messages for using water-aerosolizing devices safely.

## INTRODUCTION

Drinking water every day is important for individual health and the provision of safe water is one of the most important interventions for reducing morbidity and mortality at the population level (Cutler & Miller 2005). Reliable access to safe drinking water is regarded as one of the greatest public health achievements of the 20th century (Centers for Disease Control and Prevention 1999). During 2011–2014, 81.4% of US adults reported drinking plain^1^ water, according to the National Health and Nutrition Examination Survey (NHANES). Of these adults, 55.2% reported drinking tap water^2^ on any given day (Rosinger et al. 2018). Demographic differences in tap water use have previously been reported, with less consumption reported among minorities (Onufrak et al. 2012; Drewnowski et al. 2013; Hobson et al. 2007; Rosinger et al. 2018), younger adults (Saylor et al. 2011; Drewnowski et al. 2013), and women (Saylor et al. 2011). In the last decade, there has been an increase in bottled water consumption (U.S. Environmental Protection Agency 2019), with non-Hispanic Black and Hispanic individuals more likely to report drinking bottled water (Viscusi et al. 2015). Bottled water and public/municipal water are subject to similar health-based regulations (U.S. Environmental Protection Agency 2018; U.S. Food and Drug Administration 2019).

Most distribution systems connected to public/municipal water supplies in the United States deliver tap water that meets EPA safety standards for consumption; however, tap water is not sterile. Certain opportunistic pathogens (e.g., *Legionella*) can grow in stagnant or warm water within pipes and the root cause of illnesses associated with building water systems is often linked to poor water management (Garrison et al. 2016). Water that has undetectable levels of biofilm-associated pathogens when entering a building might become stagnant, have a reduction of disinfectant residual, or undergo temperature change, which can contribute to the proliferation of harmful pathogens. Inhalation of aerosolized tap water that contains these pathogens may cause illness in certain populations, such as older age groups or people with weakened immune systems (Centers for Disease Control and Prevention 2021a). Water-aerosolizing devices in the home that have been attributed to human illness include humidifiers (Ando et al. 2017; Woods et al. 2017; Mitting et al. 2020) and showerheads (Falkinham et al. 2008; Schoen & Ashbolt 2011; Thomson et al. 2013). Demographic data on household use of water through such devices has not previously been collected.

Waterborne disease transmission can occur in the home, yet the demographics of the people who use tap water in their homes for aerosolizing devices are unknown. The present study had two aims: (1) to quantify the consumption or inhalation of tap water in homes by adults and (2) to describe differences in use of tap water among different demographic groups, which could help for targeting awareness campaigns or identifying vulnerable communities. Using the FallStyles 2019 questionnaire, US adults were asked about their home tap water use, including their use of tap water in water-aerosolizing devices (i.e., showerheads, vaporizers, humidifiers, and continuous positive airway pressure equipment (CPAPs)).

## METHODS

### Sampling

ConsumerStyles surveys are conducted by Porter Novelli Public Services using Ipsos’ KnowledgePanel, an online panel of approximately 55,000 individuals representative of the non-institutionalized US population and recruited by mail using probability-based sampling by address (Porter Novelli 2021). Panelists are provided access to internet and tablets or laptops as needed and offered cash-equivalent rewards for survey completion. Fall ConsumerStyles, or FallStyles, was sent to 4,677 panelists ages 18 and older in October 2019. FallStyles is fielded among approximately 3,500 panelists who previously answered the spring wave. Three email reminders were sent to non-responders. Completion of the survey took approximately 33 min (median). Surveys completed in 5 min or less and incomplete surveys were removed from the dataset (*n* = 12). A total of 3,624 respondents completed the survey, yielding a 77.5% response rate. See the Porter Novelli website for more information on methodology.

### Survey question

To answer the question ‘How do you use your household tap water?’ participants were instructed to select all that apply from the following options: ‘I don’t use any tap water in my home’, ‘Drinking, rinsing produce, or making ice’, ‘I wash through use of a shower head’, ‘Filling vaporizer, humidifier, CPAP, etc.’ Two additional options (‘I drain and service my home water heater’ and ‘Rinsing contact lenses or sinuses’) were used for exploratory purposes and data are not included in this report. Twenty participants refused to answer this question and were excluded from analyses.

### Demographics

The following demographic variables and mutually exclusive categories were created: gender (male/female); age (18–29, 30–44, 45–59, 60 + years old); race/ethnicity (non-Hispanic White, non-Hispanic Black; other (non-Hispanic other or 2+ races); Hispanic of any race); education (less than high school; high school; some college; Bachelor’s degree or higher); income (<$50,000 or ≥$50,000); metropolitan status (metro/non-metro); geographic region (Northeast, Midwest, South, West). All but five respondents also reported health status (excellent, very good, good, fair, and poor).

### Analyses

Weighted analyses were conducted using eight demographic variables: gender, age, race/ethnicity, education, household income, household size, metropolitan status, and census region (Porter Novelli 2021). Weights were designed to match US Current Population Survey proportions. Survey procedures were used to assess proportions of tap water use variables by demographics. Chi-square tests were obtained to test the association of each tap water use variable with each demographic variable. *Post hoc* Wald F tests from contrast statements using the SurveyReg procedure were used to compare subdomains for demographic variables with more than two levels, when a chi-square test was significant. All analyses were performed using SAS software (version 9.4; SAS Institute), with *p* < 0.05 indicating significance.

## RESULTS

Among the 3,604 participants who reported tap water use, the median age was 56years (range = 18–94 years), and 47% of respondents were female. Respondents were non-Hispanic White (73.2%), Hispanic (10.4%), non-Hispanic Black (8.5%), and other race/ethnicity (7.9%). Overall, 35.7% were from the South, 23.1% the Midwest, 22.7% the West, and 18.5% the Northeast.

### Did not use tap water in home

A small percentage (6.6%) of individuals reported not using tap water at home (Table 1). People who reported not using tap water were more likely to be younger (aged 18–44 years, *χ*^2^ p<0.0001) and be non-Hispanic Black or Hispanic (*p*<0.0001; *p* = 0.0151).

### Drinking, rinsing produce, or making ice

Almost three-fourths (72.9%) of individuals reported using tap water for drinking, rinsing produce, or making ice. People who reported using tap water for consumption were more likely to be older (aged 45 years or older, *χ*^2^ *p* = 0.0051), non-Hispanic White or of other race (*p*<0.0001), more educated (some college or more, *p*<0.0001), have a higher income (*p*<0.0001), live in the Midwest (*p* = 0.018), and report higher-rated overall health (good to excellent, *p* = 0.0038).

### Washing through use of showerhead

The majority (80.6%) of individuals reported using tap water through a showerhead. People who reported using tap water for showering were more likely to be older (aged 45 years or older, *χ*^2^ *p* = 0.045), non-Hispanic White race (*p*<0.0001), more educated (some college or more, *p*<0.0001), and have a higher income (*p*<0.0001). Hispanic individuals more commonly reported using tap water for showering than did non-Hispanic Black individuals (Wald *p* = 0.023).

### Filling vaporizer, humidifier, CPAP, etc.

One in five (20.3%) individuals reported using tap water for filling a vaporizer, humidifier, CPAP, etc. People who reported using tap water to fill a vaporizer, humidifier, CPAP, etc., were more likely to be female (*χ*^2^ *p*<0.0001), 30–44 years old (*p* = 0.0003), non-Hispanic White or of other race (*p* = 0.0421), have at least a Bachelor’s degree (*p* = 0.0152), and live in the Midwest (*p* = 0.0049).

## DISCUSSION

Most respondents reported using household tap water for showering (80.6%) and about one in five US adults (20.3%) used household tap water to fill water-aerosolizing devices. Inhalation of aerosolized tap water from home showerheads and humidifiers has been linked to reported cases of respiratory illnesses in people with weakened immune systems (Falkinham et al. 2008; Falkinham 2011; Schoen & Ashbolt 2011; Thomson et al. 2013; Ando et al. 2017; Woods et al. 2017). Previous studies of how Americans use tap water report less consumption of tap water among minorities (Onufrak et al. 2012; Drewnowski et al. 2013; Hobson et al. 2007; Rosinger et al. 2018) and younger adults (Saylor et al. 2011; Drewnowski et al. 2013). We found similar demographic patterns and extend findings to tap water use in aerosolizing devices. Understanding which groups use tap water in aerosolizing devices is important for the development of targeted public health education materials. These materials could be used to encourage the consumption of safe tap water and increase awareness of good water management practices when using aerosolizing devices.

Exposure to tap water through a showerhead displayed similar demographic patterns to consumption. Although showering is an important hygiene behavior, harmful bacteria, such as *Legionella* (the bacteria that cause Legionnaires’ disease) can grow in stagnant water in showerheads when showers are turned off for a longer than normal period of time (Falkinham 2011). These bacteria can be aerosolized in the tap water which may cause respiratory illness when the aerosolized water vapors are inhaled by people who are older, current or former smokers, or who have weakened immune systems. Reports of pulmonary disease and exposure to opportunistic human pathogens, such as non-tuberculous mycobacteria and *Legionella* through home showerheads is an emerging area of concern (Falkinham et al. 2008; Schoen & Ashbolt 2011; Thomson et al. 2013; Mitting et al. 2020; Schumacher et al. 2020). An estimated 7.2 million waterborne illnesses occur annually in the US, with hospitalizations and deaths predominantly caused by biofilm-associated pathogens (Collieret al. 2021). Such pathogens can proliferate in biofilm buildup in stagnant water and poorly maintained building water systems and devices. However, it is important to note that the overall risk of illness from showering is low for the general population. Targeting education and other prevention measures toward people in older age groups or people with weakened immune systems may be particularly important as these groups may be most at-risk for biofilm-associated illnesses. People over 60 years old reported the greatest rates of showerhead use (82.6%) compared with other age groups. These educational efforts could increase awareness that water quality in buildings can decline and building owners can take steps to improve water quality before use. Flushing, cleaning, disinfecting, and maintaining showerheads can help reduce exposure to waterborne pathogens at home (Centers for Disease Control and Prevention 2021b). Specifically, this includes refreshing the water in the showerhead after it has not been used for longer than normal and following manufacturer instructions for cleaning showerheads to help prevent pathogens from growing within the device. In some settings, such as large buildings with complex water systems (e.g., multiple housing units with one or more centralized water heating systems or >10 storeys) building owners and managers need a water management program for the entire building to minimize the growth and transmission of waterborne pathogens in building water systems (Centers for Disease Control and Prevention 2021c). Control measures for *Legionella* can be implemented for systems and devices commonly associated with Legionnaires’ disease, such as building water systems and other devices that use tap water (Centers for Disease Control and Prevention 2021d).

Considerable demographic differences were found among groups that reported using tap water for aerosolizing devices such as vaporizers, humidifiers, and CPAPs. Individuals with the highest level of education were most likely to report using tap water for filling water-aerosolizing devices (24.1%). Females also reported significantly greater use of tap water for aerosolizing devices compared with males (23.9% vs. 16.9%). It is unclear whether these groups of individuals utilize aerosolizing devices more overall or if there are true differences between tap water use for these devices. Manufacturers’ instructions for some devices (i.e., CPAPs) warn against using tap water, but instructions for humidifiers generally do not. However, humidifiers in the home have been linked to illnesses such as hypersensitivity pneumonitis (Ando et al. 2017), cavitary *Pseudomonas* pneumonia (Woods et al. 2017), and Legionnaires’ disease (Mitting et al. 2020). Following the manufacturer’s instructions for emptying the water tanks of water-aerosolizing devices to allow for proper cleaning and drying can help reduce exposure to waterborne pathogens at home (Centers for Disease Control and Prevention 2021b). Furthermore, users could consider using distilled, previously boiled (and cooled), or water disinfected with chlorine bleach in portable humidifiers.

Overall, only 6.6% of individuals reported not using household tap water. The lowest rates of self-reported consumption of tap water were among non-Hispanic Black (61.6%) and Hispanic (62.0%) persons, individuals with less than a high school education (62.6%), those making less than $50,000 annually (62.8%), and individuals with self-reported poor health (58.3%). Historically, disparities in access to community water systems have been due to housing issues that reflect structural racial and class inequalities (Meehan et al. 2020). Disparities in tap water usage may also in part be attributed to lower perceptions of tap water safety among younger adults and racial and ethnic minorities. Hispanic and non-Hispanic Black individuals are less likely to trust the safety of their tap water (Hobson et al. 2007; Huerta-Saenz et al. 2012; Onufrak et al. 2012; Viscusi et al. 2015), which has been linked to reduced tap water drinking (Hobson et al. 2007) and increased bottled water consumption among these groups (Viscusi et al. 2015). Adults with lower socio-economic status are also more likely to report concerns over tap water safety (Onufrak et al. 2012). These concerns are often justified, as increased health-based violations of the Safe Drinking Water Act have been associated with minority populations in some communities (Fedinick et al. 2019); however, it is important to note that bottled water often contains treated municipal tap water. Local public health authorities should guide community-specific messaging based on the local conditions and promote the consumption of safe tap water among these demographic groups in the United States where appropriate.

## LIMITATIONS

Results are subject to at least five limitations. First, survey responses are self-reported from a single question asked at one point in time and without direct measures of household water use. Due to recall error and reporting bias, responses may not accurately reflect respondents’ actual tap water use. Single self-reported estimates of water consumption are similar to average repeated estimates, but repeated estimates can obtain more accurate patterns of water use, particularly if there is temporal variation. Second, while this survey is weighted to be representative of the US population, small sample sizes within subgroups can make it hard to have precise estimates for certain populations. Fourth, for the water-aerosolizing devices, we lack the ability in this dataset to restrict analyses to respondents who own such a device (i.e., vaporizer, humidifier, CPAP) and choose to use distilled water instead of tap water, limiting the utility of the comparison group who answered negatively. Fifth, respondents were asked whether they used tap water to either drink, rinse produce, or make ice. Since these uses for tap water were combined into one question, we cannot conclude that 72.9% of people are drinking water from the tap. It is possible that more people use tap water for rinsing produce or making ice, and not for drinking.

## CONCLUSION

These results point to demographic groups where future public health efforts are needed to promote consumption of safe household tap water, as well as good water management practices when using household devices that aerosolize tap water, especially for people with weakened immune systems. There is a need for future epidemiological studies to measure the relationship between water use and health in home settings.

## Figures and Tables

**Table 1 |:** Demographic characteristics of tap water use – United States, FallStyles 2019 (*N* = 3,604)

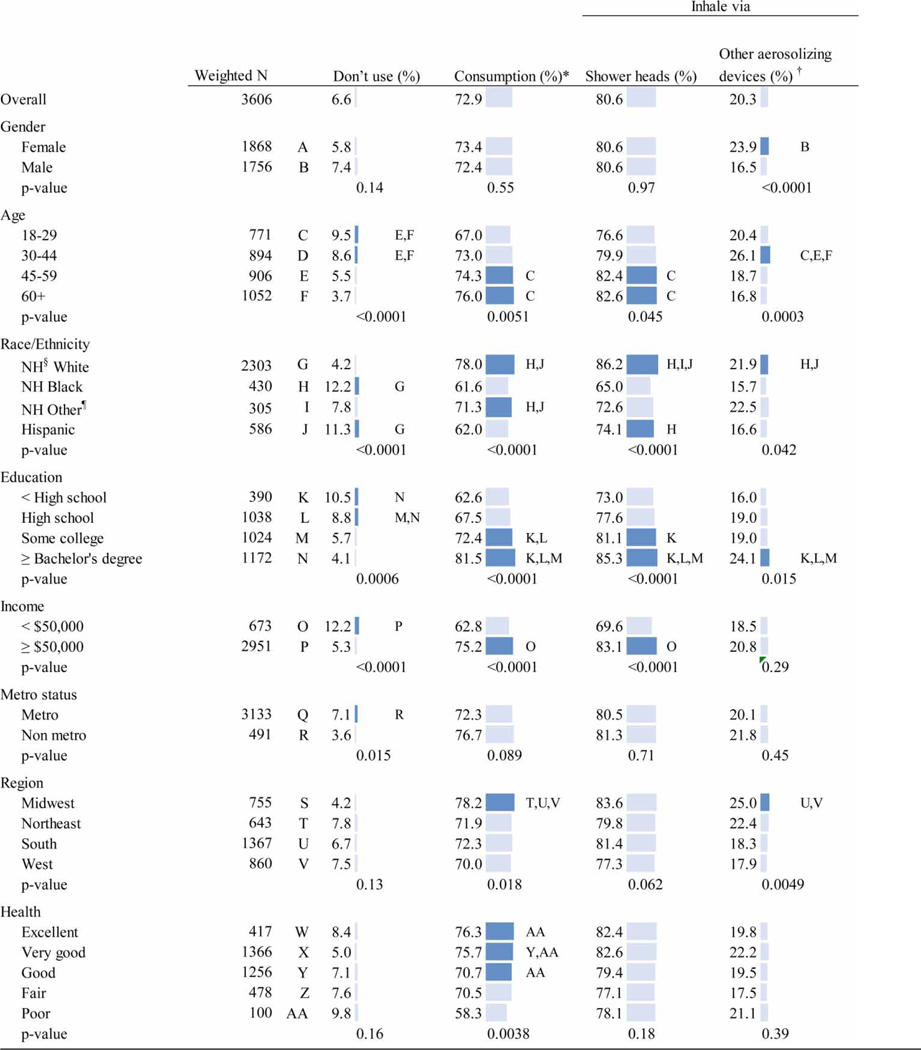

Letters indicate significant chi-square *p*-values or Wald *F*-tests where there are three or more categories.

* Consumption refers to using tap water for drinking, rinsing produce, or making ice.

† Other aerosolizing devices refer to humidifiers, vaporizers, CPAPs, etc.

§ NH, Non-Hispanic.

¶ Other race/ethnicity refers to other non-Hispanic races or 2+ races.

## Data Availability

This dataset can be licensed from Porter Novelli Public Services by contacting Deanne Weber (deanne.weber@porternovelli.com). Data cannot be made publicly available; readers should contact the corresponding author for details.

## HIGHLIGHTS

- Most individuals report using tap water through a showerhead at home, and one in five report using in aerosolizing devices, such as vaporizers and humidifiers.
- Demographic differences were found among groups that reported using tap water for aerosolizing devices.
- Public health efforts are needed to promote good water management practices when using household devices that aerosolize tap water.

## Footnotes

1 Plain water in this study was referred to as the sum of bottled and tap water consumption.

2 For the purposes of this paper, tap water is referring to water that is supplied to a tap and piped inside of homes, whether it is from a public or private source. Typical uses of tap water include drinking, washing, cooking, and flushing of toilets.
